# Aflatoxin B1 and M1: Biological Properties and Their Involvement in Cancer Development

**DOI:** 10.3390/toxins10060214

**Published:** 2018-05-24

**Authors:** Silvia Marchese, Andrea Polo, Andrea Ariano, Salvatore Velotto, Susan Costantini, Lorella Severino

**Affiliations:** 1 Unità di Farmacologia e Tossicologia—Dipartimento di Medicina Veterinaria e Produzioni Animali, Università degli Studi di Napoli “Federico II”, 80138 Napoli, Italy; silv.marchese@gmail.com (S.M.); andrea.ariano@unina.it (A.A.); velotto@unina.it (S.V.); lseverin@unina.it (L.S.); 2 Unità di Farmacologia Sperimentale, IRCCS Istituto Nazionale Tumori “Fondazione G. Pascale”, 80131 Napoli, Italy; a.polo@istitutotumori.na.it

**Keywords:** aflatoxin, AFB1, AFM1, cancer

## Abstract

Aflatoxins are fungal metabolites found in feeds and foods. When the ruminants eat feedstuffs containing Aflatoxin B1 (AFB1), this toxin is metabolized and Aflatoxin M1 (AFM1) is excreted in milk. International Agency for Research on Cancer (IARC) classified AFB1 and AFM1 as human carcinogens belonging to Group 1 and Group 2B, respectively, with the formation of DNA adducts. In the last years, some epidemiological studies were conducted on cancer patients aimed to evaluate the effects of AFB1 and AFM1 exposure on cancer cells in order to verify the correlation between toxin exposure and cancer cell proliferation and invasion. In this review, we summarize the activation pathways of AFB1 and AFM1 and the data already reported in literature about their correlation with cancer development and progression. Moreover, considering that few data are still reported about what genes/proteins/miRNAs can be used as damage markers due to AFB1 and AFM1 exposure, we performed a bioinformatic analysis based on interaction network and miRNA predictions to identify a panel of genes/proteins/miRNAs that can be used as targets in further studies for evaluating the effects of the damages induced by AFB1 and AFM1 and their capacity to induce cancer initiation.

## 1. Introduction

Aflatoxins are secondary metabolites produced by different strains of fungi, like *Aspergillus flavus* and *A. parasiticus*, widely found as contaminants in a great variety of crops—cereals, oilseeds, tree nuts and spices. Although it is well known that a hot and humid climate promotes diffusion of aflatoxin-producing moulds, representing a greater hazard in tropical areas of the world, the contamination is commonly due to the combination of meteorological conditions, environmental factors and improper agricultural practices, like incorrect harvesting and storage of crops. Indirect exposure to aflatoxins is another point of concern to human and animal health since these compounds can be transferred to offspring during gestation or lactation, or to other species upon the assumption of contaminated products like milk, eggs and meat. Accordingly, for all these reasons aflatoxins still represent a great socio-economic and health issue for both developing and industrialized countries.

Interest in aflatoxins rose in 1960’s when this class of mycotoxins was identified for the first time after an outbreak of acute feed-related mycotoxicosis occurred in England [1]. Since then, different studies ascribed to these compounds some toxic, carcinogenic, mutagenic, teratogenic and immunosuppressive effects on animals and humans [2], defining the liver as the major target organ [3]. The International Agency for Research on Cancer (IARC) evaluated both epidemiological and laboratory studies and indicated aflatoxins as carcinogenic (Group 1) and potentially carcinogenic to human (Group 2B) [4]. The wide range of adverse effects caused by aflatoxin assumption is named aflatoxicosis and has been reported in two forms: (i) “acute intoxication” caused by short exposure to great amount of toxins and characterized by severe liver damage, jaundice, haemorrhage, oedema and eventually death; and (ii) “chronic sublethal exposure,” which leads to immunosuppression, nutritional dysfunctions and cancer.

Among these toxins, Aflatoxin B1 (AFB1) is considered the most recurrent and also the most harmful. Its carcinogenicity and immunosuppression capacity have been extensively reported in all kind of animals, including poultry [5], trout [6], cattle [7] and rats [8,9] with different incidence across species, gender and age. The toxicity in humans has been assessed in association with different outbreaks of acute intoxication, especially in developing countries [10]. Many epidemiological studies focused on the connection between aflatoxins assumption through contaminated food and health problems [11,12]. Several in vitro studies demonstrated that the carcinogenicity of AFB1 is prevalently exerted upon activation by Cytochromes P450 (CYP450) in the liver and elucidated the mechanism of its toxicity [13]. Immunoresponse modulation has been observed on murine macrophages after AFB1 exposure; in fact, some authors showed an anti-proliferative action not related to apoptotic pathways and a reduction in NO levels upon exposure to not cytotoxic concentrations [14].

Another toxin causing great concern is Aflatoxin M1 (AFM1), the principal hydroxylated metabolite of AFB1, found in milk (hence the designation M) of mammals fed upon contaminated feedstuff. Carry-over of AFB1 as AFM1 in the milk of dairy cows has been established to range from 0.3% to 6.2% [15]. However, AFM1 was also found in lactating mother’s milk [16]. Several studies reported carcinogenic [9] and immunosuppressive effects [17] similar to that of AFB1, on both humans and other animals, even if with a less potent effect. Neal et al., demonstrated toxic potential of AFM1 exerted even in absence of the metabolic activation typically needed to AFB1, thus pointing out that caution should be put when classifying AFM1 as essentially detoxification product of AFB1 metabolism [13]. However, AFM1 is the only mycotoxin for which maximum residue limits (MRLs) in milk were established.

Even if a great number of studies focused on aflatoxins in the past fifty years, a complete assessment of the risk for human health has not been completed. Still few in vitro studies can be found, in particular regarding AFM1, in contrast with the concern aroused. To better understand the effects on human health, with particular regard to children which results more sensitive to intoxications due to biological and exposure causes, more studies should be carried out.

Therefore, in this review, we decided: (i) to summarize the activation pathways of AFB1 and AFM1; (ii) to describe the data, already reported in literature, about their correlation with cancer development and progression; and (iii) to identify by a bioinformatic analysis a panel of genes/proteins/miRNAs that can be used as targets in further studies for evaluating the effects of the damages induced by AFB1 and AFM1 and their capacity to induce cancer initiation.

## 2. Chemical Properties of AFB1 and AFM1

**Figure 1:**
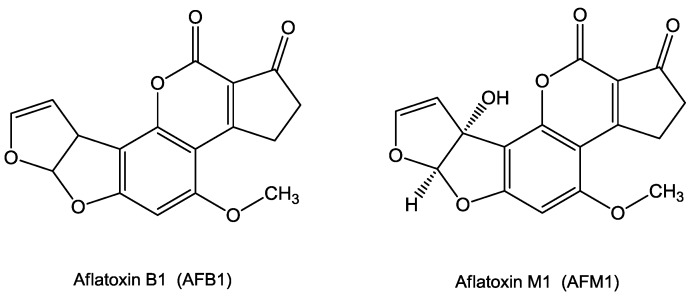
Chemical structures of Aflatoxin B1 and Aflatoxin M1.

In general, the aflatoxins are a family of compounds generally classified as difuranocoumarins, highly substituted coumarin derivatives containing a fused dihydrofurofuran moiety. In particular, AFB1 is characterized by the fusion of a cyclopentenone ring to the lactone ring of the coumarin structure (Figure 1) and by strong fluorescence emission in the blue region (hence the designation B) when exposed to ultraviolet light. AFM1 is the principal hydroxylated metabolite of AFB1 and is produced upon the action of Cytochrome P450 1A2 (CYP1A2) [18,19]. It is strongly fluorescent, emitting blue-violet light.

Both toxins have similar chemical properties: they are slightly soluble in water, insoluble in nonpolar solvents and freely soluble in polar organic solvents [20]. They have strong thermal stability, even at high temperature (>100 °C), that prevent them from being thermally degraded during food manufacturing. This represents a great obstacle in the reduction of aflatoxin food contamination, especially in milk and dairy products, since pasteurization and other thermal treatment alone showed to be poorly effective [21,22].

Other chemical properties like instability to UV light or extreme pH condition (<3 or >10) and the reactivity of lactone moiety in presence of ammonia or hypochlorite, led to the development of other methods of decontamination of feed and food. However, several physical treatments like microwaves, gamma-rays, X-rays and ultra-violet light have been investigated but controversial results discouraged the use of these methods, especially for heavily contaminated samples [23]. At present, ammoniation [24] and adsorption on clays or organic adsorbents [21] are commonly used to assure a good level of decontamination without disruption of the nutritional properties or safety of feed. Focusing on AFM1 in milk, recently some researchers are focusing on the AFM1-binding capacity of different strains of Lactobacilli [25,26].

## 3. Principal Activation Pathways of AFB1 and AFM1

**Figure 2:**
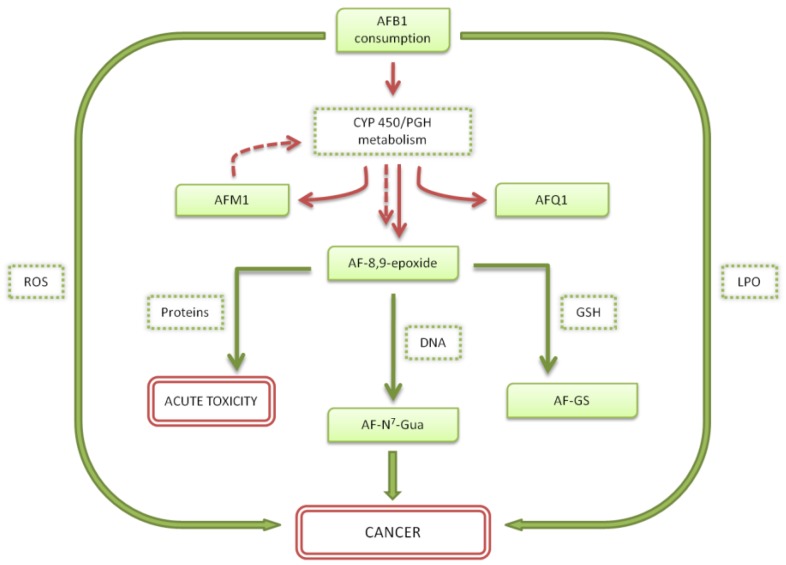
Schematic representation of AFB1 and AFM1 metabolism.

To understand the mechanism through which aflatoxins exert their toxic effects, it is important to understand how they are metabolized. The aflatoxins have been reviewed already in 1994 by Eaton and Gallagher [27]. In Figure 2 we report a schematic representation of AFB1 and AFM1 metabolism.

In detail, AFB1 is mainly metabolized in the liver upon the action of the microsomal mixed function oxidase (MFO) enzymes belonging to the superfamily of CYP450. Upon action of these oxidases, AFB1 is converted in the reactive 8,9-epoxide, existing as two stereoisomers, exo and endo, with the former reported to be the toxic species responsible for AFB1 genotoxic properties [13]. The exo-8,9-epoxide has a high binding affinity toward the DNA, forming the 8,9-dihydro-8-(N7-guanyl)-9-hydroxy-AFB1 (AFB1-N7-Gua) adduct, thus leading to DNA mutations [28]. This epoxide form is involved also in other pathways: (i) conjugation with glutathione (GSH) catalysed by Glutathione-S-Transferase (GST) with subsequent excretion as AFB-marcapturate; this pathway is vital for the detoxification of AFB1 as a carcinogen, even if a depletion of GHS could lead to high levels of reactive oxigene species (ROS) causing oxidative damage [29]; (ii) enzymatic and non-enzymatic conversion in AFB1-8,9-dihydroxydiol, that can be further converted in the dialdehyde form; aflatoxin dialdehyde can be excreted through urine as dialcohol upon action of aflatoxin aldehyde reductase (AFAR) or can bind proteins, like albumin [18]; (iii) binding to other macromolecules like proteins or RNA, causing dysregulation of normal cellular functions and inhibition of proteins, DNA and RNA synthesis [28]. Microsomal biotransformation of AFB1 includes also hydroxylation of the toxin, leading to the formation of more polar and less toxic metabolites, mainly AFM1 and Aflatoxin Q1 (AFQ1). Different studies tried to assess the role of CYP450 enzymes responsible for the formation of carcinogenic or detoxifying metabolites. CYP1A2 and CYP3A4 resulted to be the most active isoenzymes of this family and capable to activate AFB1 [30]. In detail, CYP3A4 is responsible for the formation of AFB1-exo-8,9-epoxide and of little amount of AFQ1, whereas CYP1A2 leads to both exo- and endo-8,9-epoxide and to the hydroxylated AFM1. The other two isoenzymes that resulted to have AFB1 as a substrate, even if in minor extent, are CYP3A7, expressed in human foetal liver and CYP3A5 [18].

It is well established that AFB1 epoxidation is the key step in the genotoxic process and thus in the carcinogenesis. The high affinity of the epoxide intermediate for purine bases of DNA leads to the formation of AFB1-N7-Gua adduct, that promotes mutations in nucleotide sequence. The charged adduct causes depurination and thus apurinic site formation [18]. The predominant mutation caused by AFB1-N7-Gua adduct has been identified to be the G→T transversion on the site of the original adduct [31]. Moreover, the mutation has been reported to affect specific base pair locations, showing selectivity towards guanine bases with a guanine or a cytosine as 5′ base and more specifically at the third base of codon 249 of the p53 tumour suppressor gene [32]. This mutation resulted really common in a great number of epidemiological studies on hepatocellular carcinoma (HCC) patients from regions of high aflatoxin exposure, strengthening the association between HCC incidence and aflatoxin exposure [33]. Another mutation has also been found in c-KRAS oncogene in AFB1-induced HCC in rats [34] and activation of human HRAS proto-oncogene was reported in vitro [35] suggesting an involvement of these genes in AFB1-related tumorigenesis.

Interestingly, aside from the principal biotransformation pathway involving CYP, other activation mechanisms have been reported. In fact, the epoxidation catalysed by Prostaglandin H (PGH) synthase has been described by Battista et al. [36], whereas Weng et al. hypothesized a novel mechanism in which lipid peroxidase (LPO) is the main responsible for AFB1 carcinogenesis, triggering the production of cyclic α-methyl-γ-hydroxy-1,N2-propano-dG (meth-OH-PdG) adduct and inhibiting DNA repair [37].

Another important role in DNA damage and thus carcinogenesis, is played by oxidative stress. In fact, a recent study showed that AFB1 induced ROS and oxidative stress and activated mitochondrial ROS-dependent signal pathways, which induced apoptosis through the mitochondrial signal pathway [38].

On the other hand, it is important to underline that AFM1 is primarily considered a detoxification product of AFB1 metabolism, showing only 10% of mutagenicity compared to its precursor [39]. The metabolic fate of AFM1 resulted to be similar to that of AFB1, with the difference that AFM1 represents a poorer substrate for epoxidation, thus explaining the differences in genotoxicity potencies. Moreover, it has been reported that CYP activation is not required to AFM1 to exert cytotoxic effects [13].

## 4. Aflatoxins Related Diseases

### 4.1. Liver Cancer

It is already reported in literature that the increased risk of liver cancer is correlated to AFB1 exposure. In fact, AFB1was indicated in 2002 to belong to Group 1 of the carcinogens because it induces the formation of DNA adducts that contribute to liver cancer formation [40]. AFB1 is resulted to be an important hepatocarcinogen, with 4.6–28.2% of HCC cases globally attributed to AFB1 exposure [41]. Moreover, Hepatitis B virus (HBV) can increase the risk of HCC in AFB1-exposed people by 30-fold [42]. High exposure concentrations cause acute hepatitis and, as consequence, the chronic exposure causes the development of liver cancer [43]. Recently, some authors evaluated the correlation between AFB1 exposure, expression changes of autophagy-related protein P62 and prognosis of patients with chronic HBV infection-related HCC. In detail, they focused on the Aldo-Keto Reductase Family 7 Member A3 (AKR7A3), NAD(P)H Quinone Dehydrogenase 1 (NQO1) and Nuclear factor (erythroid-derived 2)-like 2 (NRF2) levels in two groups of patients with high and low P62 expression, respectively. It is important to remember that: (i) NRF2 encodes a transcription factor that regulates genes which contain antioxidant response elements in their promoters; (ii) NQO1 encodes a cytoplasmic 2-electron reductase that prevents the one electron reduction of quinones resulting in the production of radical species; and (iii) AKR7A3 encodes an aldo-ketoreductase that is involved in the detoxification of aldehydes and ketones. These studies demonstrated that the AFB1(+)/HBV(+)group: (i) had the lowest overall survival and disease-free survival; and (ii) showed higher P62 expression correlated with a decrease of AKR7A3 expression and an increase of NRF2 and NQO1 expression. All these data evidenced that AFB1 exposure can induce an increase of P62 expression and an increased probability of cancer recurrence [44]. On the other hand, the genotoxic effects of AFB1 are still unknown and recently some authors treated the human HL7702 hepatic cells with Microcystin-LR (MC-LR) and AFB1 and demonstrated that MC-LR and AFB1 co-exposure: (i) induced DNA damage; and (ii) increased the activity of Superoxide Dismutase and Catalase, the levels of glutathione and the expression of APE1 (apurinic/apyrimidinic endonuclease 1) [45]. Considering that it is clear the correlation between AFB1 exposure and liver cancer, the researchers are studying how a diagnostic tool can be developed for early detection of the AFB1 effects. In detail, the specific mutational spectrum of the liver carcinogen AFB1 was identified in a mouse model many months before aflatoxin-induced tumours by duplex-consensus sequencing technology. Authors verified the diagnostic power of this spectrum by accurately identifying the subset of cancers associated with AFB1 exposure among the sequenced human liver tumours [46]. 

To investigate the effects of long-term exposure to AFB1 on type 1 diabetes mellitus (T1DM), some authors analysed the mice livers with T1DM after AFB1 treatment and highlighted that in the T1DM/AFB1 group the levels of the major urinary protein 1 (MUP1) were lower. Hence, considering that MUP1 is used as marker of higher insulin sensitivity, it is clear that AFB1 exposure induces the increased levels of blood glucose in mice and, also, the high probability to develop liver cancer [47]. Moreover, other studies demonstrated the importance of time intervals between AFB1 exposure and HCC development in absence and in presence of cirrhosis and HBV and evidenced that AFB1 exposure may increase in a dose-response manner the risk to develop cirrhosis and HCC in HBV patients [48].

Recently, some authors evaluated what genes are methylated and involved in the cellular transformation after AFB1 exposure. In particular, human hepatocyte cells (L02) that express an oncogenic allele of H-Ras were treated with AFB1; these cells were named L02R and L02RT-AFB1 cells, respectively.In L02RT-AFB1 cells, the authors verified that after AFB1 exposure: (i) RUNX3 (encoding a member of the runt domain-containing family of transcription factors that can function as a tumour suppressor) was methylated; (ii) LINE-1 (representing transposable elements in the DNA) was hypomethylated; (iii) the expression of CXCR4 (encoding a CXC chemokine receptor specific for stromal cell-derived factor-1), INTS1 (being a subunit of the Integrator complex, which associates with the C-terminal domain of RNA polymerase II large subunit), JUNB (encoding a transcription factor involved in regulating gene activity following the primary growth factor response), NAV1 (encoding a protein that contains coiled-coil domains and a conserved AAA domain characteristic for ATPases associated with a variety of cellular activities), POLL (encoding a DNA polymerase that plays a role in non-homologous end joining and other DNA repair processes) and RARRES1 (being a retinoid acid (RA) receptor-responsive gene and encoding a type 1 membrane protein)was decreased; and, hence, (iv) all these factors induced an increased DNA damage [49].

In general HCC tissues that are associated to AFB1 exposure present mutated ADGRB1 (adhesion G protein-coupled receptor B1) gene, capable of inducing the overexpression of PD-L1 [50].

Moreover, the effects of AFB1 on miRNA expression were investigated during the initiation phase of carcinogenesis using liver tissues and blood from F344 rats exposed to AFB1 for 4 weeks. These studies have evidenced that AFB1 up-regulated mainly miR-122-5p, 34a-5p and 181c-3p in both liver tissues and blood compared with controls suggesting that there are epigenetic changes induced by AFB1 during the initial step of carcinogenesis [51].

Also, some authors evaluated the gene expression in rat HCC tissues after or without AFB1 exposure. Anti-apoptosis genes, as well as about 2250 annotated lncRNAs, resulted in being up-regulated in the HCC sample and were involved in apoptosis regulation, DNA repair and cell cycle. Therefore, these authors concluded that AFB1 exposure induced apoptosis and expression changes in lncRNA and protein coding genes [52].

In general, in HCC patients after AFB1 exposure, there is a dysregulation of epigenetic mechanisms. In fact, after AFB1 exposure, human hepatocytes showed six up-expressed and hypomethylated genes, such as Cyclin K (CCNK), Diaphanous Related Formin 3 (DIAPH3), histone cluster 1 H2B family member f (HIST1H2BF), RAS Oncogene 27A (RAB27A), Proliferating Cell Nuclear Antigen (PCNA) and Thioredoxin Reductase 1 (TXNRD1), that can be used as early markers of HCC development correlated to AFB1 [53].

About the correlation between AFB1 and the formation of HCC cancer stem cells (CSCs) few scientific data are reported. CSCs from HepG2 cells were produced through mutations induced byAFB1 exposure demonstrating that AFB1-mediated mutations were capable of inducing the hepatic cancer cells conversion to CSCs [54].

Also, some authors evaluated the expression of miRNAs in human hepatic HepaRG cells after AFB1 treatment. It is important to remember that, under a standard nomenclature system, the names of the miRNAs are assigned to experimentally confirmed miRNAs before publication; hence, miR is followed by a dash and a number and the latter indicates the order of naming. In detail, in AFB1-treated HepaRG cells some authors demonstrated: (i) the marked over-expression of miR-410; (ii) the inhibition of the expression of miR-122 with the related HNF4A/miR-122 regulatory pathway [55]. However, another study showed that AFB1 treatment can induce in HepG2 cells the up-expression of miR-34a and the downregulation of the Wnt/β-catenin signalling pathway [56].

However, AFM1 is AFB1 metabolite that is excreted into milk and was classified by IARC as human Group 2B carcinogen.

Already in 1997, the effects of AFM1 metabolism and its capability to form DNA adduct was evaluated in HBV-associated HCC patients demonstrating a strict correlation between HBV-associated HCC and urinary AFM1 levels [57]. These data were confirmed also in a published paper in 1999 where the authors collected for ten years some chronic HBV patients and evaluated both if AFB1 exposure with the concomitant presence of Hepatitis C virus (HCV) was associated with increased HCC risk and the urine levels of AFM1 in these patients. HCC risk resulted to be increased 3.3-fold in the patients with increased urinary levels of AFM1 and in HBV presence [58]. In Egypt, in urine and serum of cirrhosis and HCC patients, the levels of AFM1 were evaluated. The results demonstrated that: (i) there is higher AFM1 concentration in serum and urine of cirrhotic than HCC patients and controls; and (ii) there is higher AFM1 concentration in urine of HCC patients from upper than lower Egypt [59].

Moreover, a longitudinal study focused on the evaluation of the relationship between primary liver cancer (PLC) initiation and AFM1 exposure was conducted in 515 patients with chronic hepatitis. It showed that PLC year incidence increased with higher urinary amount of AFM1 that is correlated with liver dysfunction [60].

In another study, the urinary AFM1 levels and the serum alpha-fetoprotein levels were evaluated. The obtained data evidenced that urinary AFM1 levels were increased in the pre-spinning, spinning and weaving sub-groups suggesting a higher risk to develop HCC [61].

### 4.2. Lung Cancer

When the workers are subjected to pulmonary AFB1 exposure due to grain dusts, they can develop lung cancer [62]. Thus, AFB1 is indicated as a pulmonary carcinogen and some different epidemiological studies focused on the association between AFB1 and lung cancers. 

In 1996, some authors investigated the AFB1 detoxification in lung tissues from patients subjected to lobectomy. Their studies demonstrated that AFB1 exposure induced the production of a DNA binding metabolite by lung cytosols. Its cytosolic activation is strictly correlated with lipoxygenase (LOX) and PGH synthase (PHS) activities and capable to increase the human pulmonary susceptibility to AFB1 [63].

Another study has demonstrated that AFB1 is bioactivated by CYP450 in lung cells from rabbits and AC3F1 (A/J В C3H/HeJ) mice. In detail, AFB1-induced AC3F1 mouse lung tumours present mutated K-ras and these mutations occur preferentially in nonciliated bronchiolar epithelial cells after AFB1 treatment but prior to tumour development. Moreover, it is important to underline that AFB1-induced mouse lung tumours present up-regulation of tumor protein p53 (TP53) protein, even if heterogeneously distributed, as well as TP53 mutations. These data are very important because TP53 encodes a tumour suppressor protein that responds to diverse cellular stresses to regulate expression of target genes and induces cell cycle arrest, apoptosis and changes in metabolism. However, these authors demonstrated also that the lung tissues from GSTM1-null individuals did not show decreased levels of AFB1 epoxide conjugation [64]. 

In 2001 the activation of AFB1 by CYP450 and the related detoxification by GST were examined in cultured normal human bronchial epithelial (NHBE) cells to understand the potential role of AFB1. In detail, these cells were treated with AFB1 for 48 h and the formation of both AFM1 and the detoxified metabolite AFQ1 was obtained after treatment with 1.5 μM of AFB1. Western immunoblots showed that CYP1A, CYP3A4 and glutathione S-transferase (GST) are constitutively expressed in NHBE cells. Expression of CYP1A and GST were significantly increased in 3-methylcholanthrene (3MC) pre-treated cells compared to CYP3A4 expression that resulted to increase lower. These authors concluded that: (i) NHBE cells activate AFB1 inefficiently but contain CYPs that are known to be responsible for AFB1metabolism; and, hence, (ii) exposure to polycyclic aromatic hydrocarbons (PAHs), such as 3MC, which induce CYP(s) and can increase the harmful effects of AFB1 exposures in human airways [65].

In another study effects of low concentrations of AFB1 on the expression of TP53 and MDM2 (encoding a nuclear-localized E3 ubiquitin ligase that can promote tumour formation by targeting tumour suppressor proteins like TP53) were highlighted in BEAS-2B, a human bronchial epithelial cell line transfected with CYP1A2 (B-CMV1A2) and CYP3A4 (B3A4) cDNA. Exposure to increasing AFB1 concentrations for 30 min resulted able to induce decreased TP53 levels mainly in B-CMV1A2 cells and increased MDM2 levels in both B-3A4 and B-CMV1A2 cells. However, AFB1 induced proteolytic caspase-3 activation in B-CVM1A2 cells treated with AFB1 suggesting the higher susceptibility of these cells to AFB1 treatment [66]. Also, another study evidenced that CYP2A13 was involved in metabolizing AFB1 to its carcinogenic/toxic AFB1-8,9-epoxide and can play a critical role in human lung carcinogenesis related to inhalation [67].

Also, oxidative DNA damages can be correlated to the AFB1 carcinogenicity on lung. In fact, AFB1 is capable to induce 8-hydroxy-2′-deoxyguanosine (8-OHdG) formation in mouse lung. To verify if treatment with polyethylene glycol-conjugated catalase (PEG-CAT) can prevent 8-OHdG formation, mouse lung tumorigenesis was analysed after female A/J mice were treated with PEG-CAT and/or AFB1. Unexpectedly, the mean number of tumours per mouse in the group treated with PEG-CAT plus AFB1 resulted greater than that of the group treated with AFB1 alone, suggesting that catalase is not able to protect against AFB1-induced mouse lung tumorigenicity [68].

AFB1 can initiate cancer also by causing oxidative DNA damage and 8-oxo-7,8-dihydro-2′-deoxyguanosine (8-oxodG) lesions that can be repaired with 8-oxoguanine glycosylase (OGG1) treatment. Therefore, some authors studied the effect of OGG1 deficiency on AFB1-induced oxidatively damaged DNA and tumorigenesis. OGG1 null mice were given a single dose of AFB1 or DMSO. AFB1-treated OGG1 null mice showed weight loss and mortality even if the lung cancer incidence did not increase. In DNA from lung tumours, the K-ras mutation pattern was inconsistent with initiation by AFB1. Hence, OGG1 status had not a significant effect on AFB1-induced DNA damage or tumorigenesis, whereas the deletion of OGG1 alleles induced an increased susceptibility to other aspects of AFB1 toxicity [69].

In 2012 the cytotoxicity and genotoxicity of AFB1 were tested on human adenocarcinoma lung cells A549. Considering the three comet parameters such as tail length, tail intensity and tail moment, AFB1 resulted to induce a significant DNA damage and a significant increase in all three micronucleus parameters compared to the control. Moreover, AFB1 provoked a statistically significant increase in the number of formed micronuclei (MN) and a slightly decreased formation of nuclear buds (NB) and nucleoplasmic bridges (NPB) [70].

Recently the effects of AFB1 on Src kinase and insulin receptor substrate (IRS) have been evaluated in lung cancer cells and cell migration was monitored. In detail, Western blot analysis was to evaluate IRS expression and Src, Akt and ERK phosphorylation in lung cancer cell lines, A549 and SPCA-1, after AFB1 treatment. These studies showed that AFB1: (i) up-regulates IRS1 and IRS2; (ii) induces Src, Akt and ERK1/2 phosphorylation; (iii) stimulates lung cancer cell migration, which can be inhibited by saracatinib [71].

In 2016 some authors investigated the role of miRNAs in the lung cancer development using P50 B-2A13 cells that are human bronchial epithelial cells expressing CYP2A13. They demonstrated that miR-138-1*: (i) can inhibit the proliferation, migration and invasion of these cells; and (ii) can decrease PDK1 expression. Therefore, miR-138-1* can affect the lung malignant transformation induced by AFB1 through PDK1 targeting [72].

Overall these data demonstrated that the correlation between AFB1 and lung cancer has been studied whereas no data are known about the possible role of AFM1.

### 4.3. Gastrointestinal Cancer and Other Cancers

In general, the digestive system represents the first barrier to carcinogens after exposure through contaminated food and for this reason, different studies focused on its interaction with the aflatoxins. In 1993, Harrison et al., suggested that human aflatoxin exposure could represent a risk to organs other than liver, even in developed countries. To prove this statement, the authors analysed normal and tumour tissues from different organs (colon, rectum, breast, cervix and liver) to find AFB1-DNA adduct. Interestingly all the samples resulted positive and, in particular, the colorectal tumour tissue showed a higher content of adduct compared to normal tissues from the same patients [73].

AFB1 effects on Caco-2 cells were evaluated alone and in association with other mycotoxins. AFB1 resulted the third more cytotoxic among tested mycotoxin showing a cytotoxic effect at the concentration of 19.28 μM [74]. In another study, HCT116 colorectal cancer cells were treated with AFB1 (3–5 μM) to verify its effect on genotoxicity and DNA damage. This study evidenced that AFB1 treatment: (i) induced a slight increase of H2AX phosphorylation in HCT116 cells by triggering ATM response; but (ii) did not activate P53, CHK1 and CHK2 and did not block G1 phase of cell cycle [75].

In 2016, some authors verified that AFB1 exposure induces MDM2 up-expression in murine intestinal cancer cells that is a negative modulator of p53. Hence, AFB1 was able to activate p53 and to block the cell cycle in S phase, but, in turn, increased MDM2 production that antagonized the p53 action [76].

AFM1 was reported to be an intestinal carcinogen already in 1987 in a study that compared the carcinogenic potency of AFB1 and AFM1. In detail, male Fisher rats were fed different doses of AFB1 and AFM1 and three intestinal adenocarcinomas, like two in the small intestine and one in the colon, were found in three mice fed the higher dose of AFM1. Cancers were not found in other AFM1 fed groups suggesting that AFM1 can have higher intestinal carcinogenicity because of retention in the digestive tract due to its higher polarity compared to AFB1 [9].

A study carried out in 2006 analysed the absorption and cytotoxicity of AFM1 on human epithelial colorectal adenocarcinoma cells, Caco-2 cells and its clone Caco-2/TC7. These authors showed that in the range of tested concentrations (0.3–32 nM) AFM1 did not cause cytotoxicity but a general cellular sufferance marked by a significant Lactate dehydrogenase (LDH) release [77]. In a similar and more recent study in which both AFM1 and AFB1 were tested on Caco-2 differentiated and undifferentiated cells, different results were obtained. In fact, at tested concentrations (0.01–1 g/mL) AFM1 resulted capable to induce cytotoxicity and concentration-dependent cell viability decrease. Moreover, LDH assay showed an increase of enzyme release, especially in differentiated cells. Moreover, these authors evaluated also ROS production and DNA damage by Comet assay and showed a more marked effect of both AFM1 and AFB1 on differentiated cells highlighting that this phenomenon is caused by the characteristic of mature enterocytes to express more metabolic enzymes and transport enzymes [78].

The same authors tested on Caco-2 cells also different combinations of mycotoxins to evaluate their possible synergic effect. In detail, AFM1 cytotoxicity was tested alone and in combinations with other three mycotoxins (ochratoxin A (OTA), zearalenone and α-zearalenol). The results evidenced that: (i) AFM1 and OTA were more cytotoxic than other toxins and (ii) AFM1 cytotoxicity increased when other mycotoxins were co-present indicating the need to re-assign the established MRLs for AFM1 considering that is very frequently found together with other mycotoxins in baby food which contains milk and cereals [79].

Recently the effects of AFM1 and OTA were evaluated also on human intestinal epithelial permeability and the mechanisms behind intestinal damage. In detail, the authors reported that in differentiated Caco-2 cells AFM1 (0.12 and 12 μM) individually or collectively with OTA increased epithelial permeability. Furthermore, this study reported after AFM1 exposure the loss of tight junction (TJ) proteins that regulate the intestinal barrier permeability and integrity. The evaluation of the levels of TJ proteins showed significantly lower expression of Occludin and ZO-1 in Caco-2 cells treated with AFM1 alone or in combination; in contrast, claudin-3 and claudin-4 levels were not significantly altered by AFM1 alone. The evaluation of TJ proteins localization by immunofluorescent staining evidenced that: (i) claudin-3 and ZO-1 were hardly detected in cells after AFM1 treatment alone and in combination, without change in cellular localization, suggesting that the protein synthesis was affected; (ii) a discontinuous cobblestone pattern and a faint immunofluorescent signal was detected also for claudin-4; (iii) the localization of occludin is not affected by AFM1 and OTA alone; and (iv)the localization of occludin is affected by AFM1 and OTA combination demonstrating these mycotoxins reduce the barrier Caco-2 properties and alternate TJ proteins distribution and levels [80].

Only few data are reported on the involvement of AFB1 but not of AFM1 to develop other cancers. Already in 1969 and 1973, a significant incidence of renal carcinomas was noted in male Wistar rats fed diets containing different amount of AFB1. Renal epithelial neoplasias, histologically similar to human kidney adenocarcinoma, were found in over one-half of the animals ingesting the highest level of AFB1 [81,82].

In 1992, MCF-7 breast cancer cells were transfected with a mammalian cell expression plasmid containing CYP450IIIA7 complementary DNA. In this way, three cell lines, termed M13, M21 and M27, which expressed the CYP450IIIA7, were obtained with a very high sensitivity against AFB1 compared to parental MCF-7 cells [83]. More recently, it has been evaluated the effect of AFB1 on cell growth and cell cycle progression in a human breast cancer cell line, MCF-7. The obtained results showed that AFB1 was cytotoxic and affected MAPK pathways [84]. Interestingly, breast cancer resistance proteins (Bcrp1/BCRP), reported to be significantly expressed in MCF-7 and BT20 cells [85], resulted able to bind AFB1 in vitro and in vivo studies, to block AFB1 accumulation in tissues expressing these proteins and to induce a higher secretion in milk [86].

Since AFB1 exposure can occur not only after inhalation and ingestion but also through skin contact, some authors evaluated the effects of AFB1 exposure on mouse. They showed that single AFB1 applications plus 12-tetradecanoyl phorbolmyristate acetate applications for 13 months were able to induce skin cancer development [87].

Recently, some authors investigated if there was a correlation between high AFB1 exposure and gallbladder cancer. Hence, they evaluated: (i) the levels of AFB1-lysine adduct in plasma samples of patients with gallstones, gallbladder cancer and gallstones without cancer; and (ii) the R249S mutation in TP53 that is associated with AFB1 exposure in tumour tissues from gallbladder cancer patients. This case-control study showed that exposure to AFB1 is associated with gallbladder cancer even if R249S mutation in TP53 is not present in the collected gallbladder cancer patients [88]. 

In 2013, a study was conducted to assess if aflatoxins contamination of wheat flour could be related to high-risk oesophageal cancer in the Golestan region of Iran. These authors demonstrated that in samples collected in oesophageal cancer high-risk areas there were higher levels of AFB1 compared to low-risk areas of the same region [89].

Finally, AFB1 exposure was associated also with gastric cancer development. A case-control study evidenced that: (i) AFB1 intake was higher in gastric cancer patients than in controls and (ii) a genetic polymorphism of CYP1A2 (rs2470890), one of the enzymes that metabolize AFB1, showed a lower risk of gastric carcinogenesis [90].

## 5. Analysis of Genes/Proteins Modulated by AFB1/AFM1 by Systems Biology Approach

On the basis of the data reported above, there are some proteins modulated by AFM1/AFB1 like CYP1A2, CYP1A5, CYP3A4, TP53, GSMT1, MDM2, CAT, OGG1, IRS1, IRS2, SRC, AKT1, MAPK1, MAPK3 and PDK1. These proteins are involved in important metabolic pathways such as FoxO signalling pathway, PI3K-Akt signalling pathway, AMPK signalling pathway, MAPK signalling pathway and VEGF signalling pathway (Table 1).

**Figure 3:**
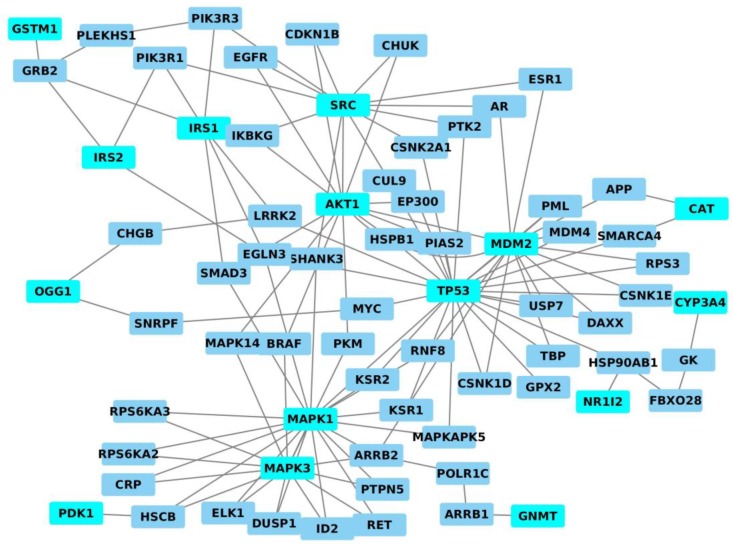
Interaction network of proteins modulated by AFM1 where these proteins are depicted in light-blue whereas the linking nodes in blue.

Considering that it is important to understand the molecular mechanisms through which these aflatoxins can modulate contemporaneously many proteins, we mapped them on the entire human interactome (INTACT) and constructed the relative interaction network by Cytoscape in order to know if and how these proteins are correlated between them using the same protocol that our group has recently developed [91]. The obtained network resulted to be composed of 71 nodes (proteins) and 121 edges (interactions). In particular, thirteen proteins modulated by AFM1/AFB1 (CYP3A4, TP53, GSMT1, MDM2, CAT, OGG1, IRS1, IRS2, SRC, AKT1, MAPK1, MAPK3 and PDK1) were present. In detail, we can underline that: (i) AKT1 shows a direct interaction with SRC and MDM2 nodes; (ii) TP53 displays a direct interaction with MAPK1 and MDM2 nodes; and (iii) MAPK1 is directly linked to MAPK3. On the other hand, the other proteins (CYP3A4, GSMT1, CAT, OGG1, IRS1, IRS2, SRC and PDK1) are correlated between them by other nodes present in the network (Figure 3). Among these nodes, we found NR1I2 (encoding a transcriptional regulator of CYP3A4) and GNMT (encoding an enzyme that catalyses the conversion of S-adenosyl-l-methionine to S-adenosyl-l-homocysteine and sarcosine) that are known to be modulated by AFM1 even if in non-specific cancer studies. In fact, AFM1 is able to increase the activity of NR1I2 protein [92], whereas GNMT induces the increased secretion of AFM1 [93]. However, in general, GNMT is known to have a role in the methionine metabolism and in the gluconeogenesis and to act as tumour gene. In fact, studies have demonstrated decreasing levels of GNMT in both HCC cell lines and tumour tissues [94] and higher cytoplasmic levels correlated to growing promotion and progression of prostate cancer [95]. On the other hand, NR1I2 has higher expression levels in prostate cancer and breast cancer progression [96] and has also a role in endometrial tumours and their progression [97] and in hematologic cancers such as B cell lymphoma [98]. It is resulted also to be involved in ovarian cancer and colorectal cancer initiation; in fact, its activation induces both tumour progression and chemotherapy resistance in both these tumours [97,99,100].

In summary, the correlation between these nodes indicates that they are strictly correlated between them at functional level and, thus, we can suggest evaluating their levels to predict the effects of AFB1 and AFM1 exposure on cancer initiation and progression.

**Figure 4:**
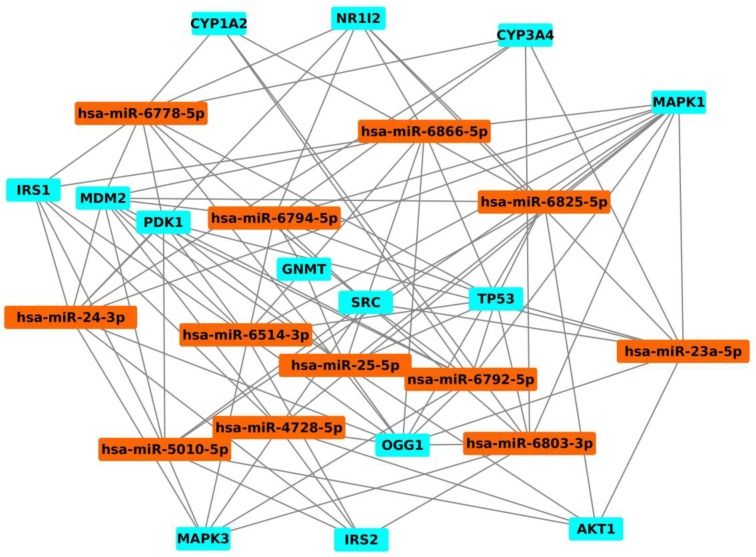
Sub-network of genes modulated by AFB1/AFM1 and twelve miRNAs linking these genes. In detail, miRNAs are shown in orange and genes in blue.

Considering the important role of miRNAs in cancer initiation and progression, we have analysed what miRNAs can target fifteen selected genes and created the related miRNA-gene interaction network using the same protocol reported in [91]. In detail, firstly, the list of miRNAs able to target the selected genes was extracted by MirNet tool; and secondly, an interaction network between selected miRNAs and genes was constructed by the Cytoscape package. Our analysis has identified 2053 miRNAs able to target fifteen genes (CYP1A2, CYP3A4, NR1I2, GNMT, NR1I2, TP53, MDM2, CAT, OGG1, IRS1, IRS2, SRC, AKT1, MAPK1, MAPK3 and PDK1). Starting from the list of all the identified miRNAs we constructed an interaction network composed by 2068 nodes and 19218 interactions. Focusing on the miRNAs able to target different genes at the same time, we evidenced that the genes are targeted by twelve following miRNAs: hsa-miR24-3p, hsa-miR-6778-5p, hsa-miR-6514-3p, hsa-miR-5010-5p, hsa-miR-23a-5p, hsa-miR-25-5p, hsa-miR-6792-5p, hsa-miR-6866-5p, hsa-miR-4728-5p, hsa-miR-6825-5p, hsa-miR-6803-3p, hsa-miR-6794-5p (Table 2 and Figure 4).

Some of these twelve miRNAs are already reported as involved in cancer in literature. In fact, it is important to underline that hsa-miR-24-3p is up-regulated in gastric cancer and breast cancer and promotes cell growth, apoptosis and invasion mechanisms [101,102]. Hsa-miR-23a-5p promotes the cell growth in gastric cancer [103], regulates colon cancer metastasis and induces invasion and progression [104], is capable to induce higher vascular and endothelial permeability in lung cancer [105] and contributes to metastasis and autophagic process in melanoma cancer [106]. In regard to hsa-miR-25-5p, it is involved in proliferation and invasion mechanisms in lung cancer [107], is associated with a poor survival in gastric cancer patients [108], regulates apoptosis in ovarian cancer [109], promotes cell proliferation in triple negative breast cancer [110] and reduces the cell invasion in prostate cancer [111]. Hsa-miR-4728-5p is a negative regulator of MAPK correlated with a negative overall survival in breast cancer [112] and was found as a tumour suppressor in the ulcerative colitis associated with colon cancer [113]. Hsa-miR-6803 results a potential diagnostic and prognostic biomarker in colorectal cancer and its increased levels are associated with a poor overall survival and prognosis [114]. No information about the involvement of hsa-miR-6778-5p, hsa-miR-6514-3p, hsa-miR-5010-5p, hsa-miR-6792-5p, hsa-miR-6866-5p, hsa-miR-6825-5p, hsa-miR-6794-5p in cancer can be found in literature, suggesting the necessity of further studies to elucidate their role in cancer initiation, also due to AFM1 and AFB1 exposure.

## 6. Conclusions

In this review, we summarize the chemical properties of AFB1 and AFM1, their activation pathways and the data, already reported in literature, about their correlation with cancer development and progression. In the context, the published data evidenced that AFB1 and AFM1 exposure were already associated mainly with liver, lung and colon cancer initiation and progression. Still few data are known about other cancers, and, hence, further studies will be necessary. 

Moreover, considering that few data suggest what genes/proteins/miRNAs can be used as damage markers due to AFB1 and AFM1 exposure, we performed an interaction network analysis and miRNA predictions able to target genes modulated by these two toxins. The network analysis suggests that thirteen proteins modulated by AFM1/AFB1 (CYP3A4, TP53, GSMT1, MDM2, CAT, OGG1, IRS1, IRS2, SRC, AKT1, MAPK1, MAPK3 and PDK1) are correlated between them both directly and through other nodes, such as NR1I2 and GNMT. On the other hand, the analysis of miRNA prediction has evidenced that there are twelve miRNAs able to target the genes known to be modulated by AFM1.

In conclusion, we suggest that it would be very interesting to focus further experimental studies on the evaluation of the expression changes of these genes/proteins/miRNAs to verify if they can be used as damage markers due to AFB1 and AFM1 exposure and, hence, as indexes of cancer initiation or progression.

## Figures and Tables

**Table 1:** Pathways in which the proteins modulated by AFB1/AFM1 are involved.

Pathway	Proteins
Linoleic acid metabolism	CYP1A2, CYP3A4
Steroid hormone biosynthesis	CYP1A2, CYP3A4
Retinol metabolism	CYP1A2, CYP3A4
Drug metabolism—cytochrome P450	CYP1A2, CYP3A4
Metabolism of xenobiotics by cytochrome P450	CYP1A2, CYP3A4
Chemical carcinogenesis	CYP1A2, CYP3A4
Nuclear Receptors in Lipid Metabolism and Toxicity	CYP3A4, NR1I2
FoxO signalling pathway	AKT1, MDM2, CAT, IRS1, IRS2, MAPK1, MAPK3
Thyroid hormone signalling pathway	AKT1, MDM2, SRC, MAPK1, MAPK3, TP53
Neutrophin signalling pathway	AKT1, IRS1, IRS2, MAPK1, MAPK3, TP53
Insulin signalling pathway	AKT1, IRS1, IRS2, MAPK1 MAPK3
mTOR signalling pathway	AKT1, IRS1, MAPK1, MAPK3
VEGF signalling pathway	AKT1, SRC, MAPK1, MAPK3
cGMP-PKG signalling pathway	AKT1, IRS1, IRS2, MAPK1, MAPK3
Prolactin signalling pathway	AKT1, SRC, MAPK1, MAPK3
PI3K-Akt signalling pathway	AKT1, MDM2, IRS1, IRS2, MAPK1, MAPK3, TP53
ErbB signalling pathway	AKT1, SRC, MAPK1, MAPK3
HIF-1 signalling pathway	AKT1, MAPK1, MAPK3, PDK1
Oestrogensignalling pathway	AKT1, SRC, MAPK1, MAPK3
Sphingolipid signalling pathway	AKT1, MAPK1, MAPK3, TP53
Platelet activation	AKT1, SRC, MAPK1, MAPK3
Aldosterone-regulated sodium reabsorption	IRS1, MAPK1, MAPK3
Chemokine signalling pathway	AKT1, SRC, MAPK1, MAPK3
Regulation of lipolysis in adipocytes	AKT1, IRS1, IRS2
Focal adhesion	AKT1, SRC, MAPK1, MAPK3
Rap1 signalling pathway	AKT1, SRC, MAPK1, MAPK3
Fc gamma R-mediated phagocytosis	AKT1, MAPK1, MAPK3
MAPK signalling pathway	AKT1, MAPK1, MAPK3, TP53
Gap junction	SRC, MAPK1, MAPK3
GnRH signalling pathway	SRC, MAPK1, MAPK3
T cell receptor signalling pathway	AKT1, MAPK1, MAPK3
TNF signalling pathway	AKT1, MAPK1, MAPK3
Toll-like receptor signalling pathway	AKT1, MAPK1, MAPK3
AMPK signalling pathway	AKT1, IRS1, IRS2
cAMP signalling pathway	AKT1, MAPK1, MAPK3

CYP: Cytochrome P450, TP53: tumor protein p53, GSMT1: Glutathione S-transferase Mu 1, MDM2: Mouse double minute 2 homolog, CAT: catalase, OGG1: 8-Oxoguanine glycosylase, IRS: Insulin receptor substrate, SRC: Proto-Oncogene Tyrosine-Protein Kinase, AKT1: serine-threonine protein kinase 1, MAPK: mitogen-activated protein kinase, PDK1: pyruvate dehydrogenase kinase 1, AMPK: AMP-activated protein kinase.

**Table 2:** List of twelve miRNAs linking and targeting genes modulated by AFB1/AFM1.

miRNA	Gene
hsa-miR24-3p	CYP3A4, IRS1, IRS2, MAPK1, MAPK3, MDM2, NR1I2, OGG1, PDK1
hsa-miR-6778-5p	CYP1A2, CYP3A4, IRS1, MDM2, NR1I2, OGG1, PDK1, SRC, TP53
hsa-miR-6514-3p	GNMT, IRS1, IRS2, MAPK1, MDM2, OGG1, PDK1, TP53
hsa-miR-5010-5p	AKT1, IRS1, IRS2, MAPK1, MAPK3, MDM2, PDK1, SRC
hsa-miR-23a-5p	AKT1, CYP3A4, MAPK1, NR1I2, OGG1, PDK1, SRC, TP53
hsa-miR-25-5p	AKT1, GNMT, MAPK1, MDM2, OGG1, PDK1, SRC, TP53
hsa-miR-6792-5p	CYP1A2, MAPK1, MAPK3, MDM2, OGG1, PDK1, SRC, TP53
hsa-miR-6866-5p	GNMT, IRS1, MAPK1, MDM2, NR1I2, OGG1, SRC, TP53
hsa-miR-4728-5p	AKT1, IRS1, IRS2, MAPK1, MAPK3, MDM2, OGG1, SRC
hsa-miR-6825-5p	AKT1, CYP1A2, MAPK1, MDM2, NR1I2, OGG1, SRC, TP53
hsa-miR-6803-3p	CYP1A2, CYP3A4, IRS2, MAPK1, MAPK3, OGG1, SRC, TP53
hsa-miR-6794-5p	CYP3A4, GNMT, MAPK1, MAPK3, MDM2, NR1I2, SRC, TP53
